# TFEB Gene Promoter Variants Effect on Gene Expression in Acute Myocardial Infarction

**DOI:** 10.3389/fcell.2021.630279

**Published:** 2021-02-25

**Authors:** Jie Zhang, Yexin Zhang, Xiaohui He, Shuai Wang, Shuchao Pang, Bo Yan

**Affiliations:** ^1^Department of Medicine, Shandong University School of Medicine, Jinan, China; ^2^Shandong Provincial Key Laboratory of Cardiac Disease Diagnosis and Treatment, Affiliated Hospital of Jining Medical University, Jining Medical University, Jining, China; ^3^The Center for Molecular Genetics of Cardiovascular Diseases, Affiliated Hospital of Jining Medical University, Jining Medical University, Jining, China; ^4^Shandong Provincial Sino-US Cooperation Research Center for Translational Medicine, Affiliated Hospital of Jining Medical University, Jining Medical University, Jining, China

**Keywords:** acute myocardial infarction, autophagy, TFEB, promoter, genetics

## Abstract

Autophagy is involved in many physiological processes. Transcription factor EB (TFEB) is a master regulator of autophagy and coordinates the expression of autophagic proteins, lysosomal hydrolases, and lysosomal membrane proteins. Though autophagy has been implicated in several human diseases, little is known regarding TFEB gene expression and regulation in the process. Since dysfunctional autophagy plays critical roles in acute myocardial infarction (AMI), dysregulated TFEB gene expression may be associated with AMI by regulating autophagy. In this study, the TFEB gene promoter was genetically and functionally analyzed in AMI patients (*n* = 352) and ethnic-matched controls (*n* = 337). A total of fifteen regulatory variants of the TFEB gene, including eight single-nucleotide polymorphisms (SNPs), were identified in this population. Among these, six regulatory variants [g.41737274T>C (rs533895008), g.41737144A>G, g.41736987C > T (rs760293138), g.41736806C > T (rs748537297), g.41736635T > C (rs975050638), and g.41736544C > T] were only identified in AMI patients. These regulatory variants significantly altered the transcriptional activity of the TFEB gene promoter. Further electrophoretic mobility shift assay revealed that three of the variants evidently affected the binding of transcription factors. Therefore, this study identified novel TFEB gene regulatory variants which affect the gene expression. These TFEB gene regulatory variants may contribute to AMI development as a rare risk factor.

## Introduction

Autophagy is one of the major digestive systems in cells. There are three subtypes of autophagy, macroautophagy, microautophagy, and chaperone-mediated autophagy. Macroautophagy (hereafter referred to as autophagy) degrades cytoplasmic macromolecules and organelles by delivering them to lysosomes. Autophagy has been involved in many physiological processes, including lipid metabolism and inflammation. Dysfunctional autophagy has been implicated in a wide range of human diseases, including cardiovascular diseases (Gatica et al., 2015; Bravo-San Pedro et al., 2017). However, the genetic causes for autophagic dysfunction and underlying molecular mechanisms remain largely unknown.

Transcription factor EB (TFEB) belongs to the MiT-TFE family of basic helix–loop–helix leucine–zipper transcription factors, which include TFEB, TFE3, TFEC, and microphthalmia-associated transcription factor (MITF). TFEB regulates several cellular processes, including lysosome biogenesis, cellular energy homeostasis, autophagy, mitochondrial turnover, innate immune response, and inflammation. Many studies have demonstrated that TFEB has been involved in the co-regulation between lysosome, autophagy, and lipid metabolism (Sardiello et al., 2009; Settembre et al., 2011, 2013; Settembre and Ballabio, 2014; Napolitano and Ballabio, 2016; Brady et al., 2018). At the transcriptional level, TFEB functions as a master regulator of autophagy and coordinates the expression of lysosomal hydrolases, lysosomal membrane proteins, and autophagy proteins. TFEB binds to CLEAR (coordinated lysosomal expression and regulation) motif, a 10 base E-box-like sequence (GTCACGTGAC), within the promoters of the autophagic and lysosomal genes (Sardiello et al., 2009; Bajaj et al., 2019).

Recent studies suggest that TFEB also controls vascular development by regulating the proliferation of endothelial cells (Doronzo et al., 2019). Overexpression of the TFEB gene in endothelial cells in mice increases angiogenesis and improves blood flow recovery after ischemic injury (Fan et al., 2018). Animal experiments show that TFEB inhibits endothelial cell inflammation and reduces atherosclerosis (Lu et al., 2017; Song et al., 2019). Thus, altered TFEB level may contribute to cardiovascular diseases. In this study, we first identified regulatory variants in the TFEB gene promoter in patients with acute myocardial infarction (AMI) and then functionally analyzed the effect of the variants on TFEB gene expression. Furthermore, the molecular mechanisms by which the regulatory variants effect TFEB gene expression were also explored.

## Materials and Methods

### Study Participants

AMI patients (*n* = 352; male 267 and female 85) were recruited from the Division of Cardiology, Affiliated Hospital of Jining Medical University (Jining, Shandong, China) during the period from March 2015 to June 2017. AMI patients were diagnosed according to clinical manifestations, electrocardiograms, elevated biochemical markers of myocardial necrosis, or coronary angioplasty. Ethnically matched controls (*n* = 337; male 167 and female 170) were recruited from Physical Examination in the same hospital during the same time period. The controls with a familial history of CAD and other heart diseases were excluded. This study was conducted in accordance with the Declaration of Helsinki (1964). The study protocol was approved by the Human Ethics Committee of the Affiliated Hospital of Jining Medical University. Written informed consent was obtained from all participants.

### Direct DNA Sequencing

Fasting venous blood was collected, and peripheral leukocytes were isolated with the Human Leukocyte Isolation system (Haoyang Biological Products Technology Co., Ltd., Tianjin, China). Genomic DNAs were extracted with the QIAamp DNA Mini kit (Qiagen, Inc., Valencia, CA, United States). The promoter region of the human TFEB gene were generated with PCR and directly sequenced. Two overlapped DNA fragments, 705 bp (−1312 ∼−608 bp) and 801 bp (−657 bp ∼ +144 bp), were overlapped, covering the TFEB gene promoter region. The PCR primers were designed using the human TFEB genomic sequence (National Center for Biotechnology Information GenBank accession no. NC_000006.12). PCR products were directly and bi-directionally sequenced on a 3500XL genetic analyzer (Thermo Fisher Scientific, Inc., Waltham, MA, United States) by Sangon Biotech Co., Ltd. (Shanghai, China). DNA sequences were then compared with the wild-type TFEB gene promoter using the DNAMAN program (Version 5.2.2, Lynnon BioSoft, Quebec, Canada), and regulatory variants including single-nucleotide polymorphisms (SNPs) were identified. Wild and variant TFEB gene promoters were analyzed using TRANSFAC and JASPAR programs to predict the binding sites for the transcription factor affected by regulatory variants.

### Functional Analysis of Regulatory Variants by Dual-Luciferase Reporter Assay

Wild-type and variant TFEB gene promoters (1386, −1309 ∼ +77 bp) were generated by PCR, which were then inserted into the KpnI and HindIII sites of a luciferase reporter vector (pGL3-basic, Promega Corporation, Madison, WI, United States) to generate expression constructs. The designated expression constructs were transiently transfected into cultured HEK-293 [CRL-1573; American Type Culture Collection, Manassas, VA, United States (ATCC), Manassas, VA, United States] and H9c2 cells (rat cardiomyocyte line; CRL-1446; ATCC), and dual-luciferase activity was examined using Dual-Luciferase^®^ Reporter Assay on a Glomax 20/20 luminometer (Promega Corporation, Madison, WI, United States). TFEB gene promoter activity was expressed as the ratio of luciferase activity over Renilla luciferase activity. Activity of the wild-type TFEB gene promoter was set as 100%, and the activity of the variant TFEB gene promoter was calculated. Transfection experiments were repeated three times independently, in triplicate.

### Prediction of Binding Sites for Transcription Factors

The TFEB gene promoter was analyzed using TRANSFAC and JASPAR programs to predict whether regulatory variants identified in AMI patients change the putative binding sites for transcription factors.

### Electrophoretic Mobility Shift Assay

To examine the effects of TFEB gene regulatory variants on the binding sites for transcription factors, electrophoretic mobility shift assay (EMSA) was conducted using the LightShift^®^ Chemiluminescent EMSA kit (Thermo Fisher Scientific, Inc., Waltham, MA, United States). Biotinylated double-stranded oligonucleotides (30 bp) containing regulatory variants were used as probes. Nuclear extracts from HEK-293 and H9c2 cells were prepared using NE-PER^®^ Nuclear and Cytoplasmic Extraction Reagent kit (Thermo Fisher Scientific, Inc., Waltham, MA, United States). Protein concentrations were determined using the Bradford protein assay. DNA-protein binding reactions were conducted for 20 min at room temperature with equal amounts of probes (0.2 pMol) and nuclear extracts (3.0 μg). The reaction mixtures were subsequently separated on a 6% polyacrylamide gel and transferred onto a nylon membrane (Thermo Fisher Scientific, Inc., Waltham, MA, United States). The oligonucleotides were cross-linked to the membrane using the UV Stratalinker 1800 (Stratagene; Agilent Technologies, Inc., Santa Clara, CA, United States) and were detected by chemiluminescence using the LightShift^®^ Chemiluminescent EMSA kit (Thermo Fisher Scientific, Inc., Waltham, MA, United States).

### Statistical Analysis

Quantitative data are expressed as the means ± standard error of the mean and were analyzed by student *t*-test using two-way analysis of variance followed by Dunnett test. The frequency of regulatory variants was compared between AMI patients and controls with χ^2^ test using SPSS v22.0 software (SPSS, Inc., Chicago, IL, United States). *P* < 0.05 was considered as statistically significant.

## Results

### Clinical and Biochemical Characteristics

This study included 689 participants, including 352 AMI patients and 337 controls. Clinical and biochemical characteristics are summarized in Table 1. Age, body mass index (BMI), triglyceride (TG), total cholesterol (TC), high-density lipoprotein cholesterol (HDL), and low-density lipoprotein cholesterol (LDL) were expressed as mean ± standard deviation. The prevalence of traditional risk factors including male sex, hypertension, diabetes, and smoking was significantly higher in AMI patients compared to controls (*P* < 0.01). TG, TC, HDL, and LDL levels in AMI patients were significantly lower compared to controls (*P* < 0.01), probably due to application of lowering-lipid medicines in AMI patients. In addition, there was no significant difference of BMI between AMI patients and controls (*P* > 0.05).

**TABLE 1:** Clinical and biochemical characteristics of AMI patients and controls^†^.

	Controls (*n* = 337)	AMI (*n* = 352)	*P*-value
Age (years, mean ± SD)	51.25 ± 12.28	61.29 ± 12.01	<0.01
Male (*n*, %)	167 (49.55%)	267 (75.85%)	<0.01
Hypertension (*n*, %)	63 (18.69%)	122 (34.66%)	<0.01
Diabetes (*n*, %)	12 (3.56%)	68 (19.32%)	<0.01
Smoking (*n*, %)	24 (7.12%)	189 (53.69%)	<0.01
BMI (kg/M^2^)	25.12 ± 3.51	25.02 ± 3.73	0.783
TG (mmol/L)	1.74 ± 0.98	1.32 ± 1.00	<0.01
TC (mmol/L)	4.88 ± 0.92	4.32 ± 1.13	<0.01
HDL (mmol/L)	1.24 ± 0.25	1.10 ± 0.26	<0.01
LDL (mmol/L)	2.95 ± 0.85	2.62 ± 0.84	<0.01

*BMI, body mass index; TG, triglyceride; TC, total cholesterol; HDL, high-density lipoprotein cholesterol; LDL, low-density lipoprotein cholesterol. ^†^quantitative data including age, BMI, TG, TC, HDL, and LDL were expressed as M ± SD.*

### Identified Regulatory Variants in the TFEB Gene Promoter

**FIGURE 1:**
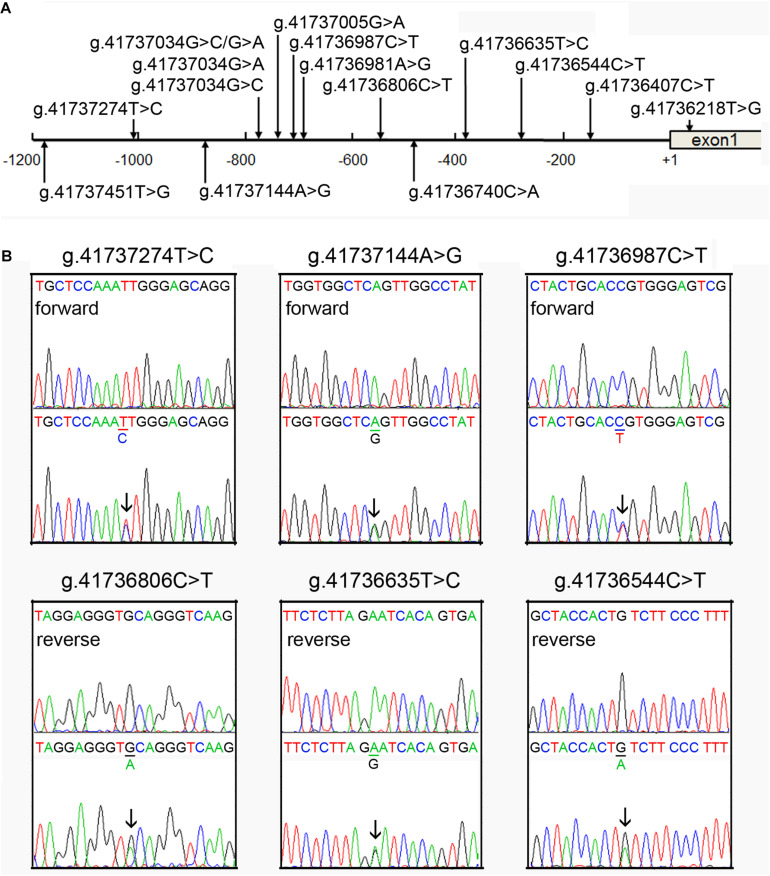
Identified regulatory variants of TFEB gene. **(A)** Locations of the regulatory variants in the TFEB gene promoter. The numbers represent the genomic DNA sequences of the human TFEB gene (Genbank accession number NC_000006.12). The transcription start site is at the position of 41736259 (+1) in the first exon. **(B)** Sequencing chromatograms of the regulatory variants identified in AMI patients. Sequence orientations are marked. Top panels show wild-type and bottom heterozygous DNA sequences. Arrows indicate the heterozygous variant.

A total of fifteen regulatory variants of TFEB gene were identified in this study population, including eight SNPs and seven novel variants. Frequency and locations of the regulatory variants are presented in Figure 1A and summarized in Table 2. Two novel heterozygous variants (g.41737144A > G and g.41736544C > T) and four SNPs [g.41737274T > C (rs533895008), g.41736987C > T (rs760293138), g.41736806C > T (rs748537297), and g.41736635T > C (rs975050638)] were only identified in six male AMI patients (Figure 1B). All the six cases were male, and the age was from 52 to 81 years old. Clinically, four AMI cases suffered from acute inferior myocardial infarction, and two from acute anterior myocardial infarction. Three of the six AMI cases are accompanied with hypertension. Four of the six AMI cases had history of smoking. None of the six cases had diabetes. In addition, the clinical and biochemical parameters of the six AMI cases are listed on Table 3.

**TABLE 2:** Regulatory variants of TFEB gene in AMI patients and controls.

Regulatory variants	Genotypes	Location†	Controls (*n* = 337)	AMI (*n* = 352)	*P*-value
g.41737451T > G	TG	−1192 bp	1	0	–
g.41737274T > C (rs533895008)	TC	−1015 bp	0	1	–
g.41737144A > G	AG	−885 bp	0	1	–
g.41737034G > C (rs73733015)	GG	−775 bp	209	196	0.239
	GC		111	136	
	CC		17	20	
g.41737034G > A (rs73733015)	GA	−775 bp	1	1	1
g.41737034G > C/G > A (rs73733015)	CA	−775 bp	1	0	–
g.41737005G > A (rs149166358)	GA	−746 bp	1	0	–
g.41736987C > T (rs760293138)	CT	−728 bp	0	1	–
g.41736981A > G	AG	−722 bp	1	0	–
g.41736806C > T (rs748537297)	CT	−547 bp	0	1	–
g.41736740C > A	CA	−481 bp	1	1	1
g.41736635T > C (rs975050638)	TC	−376 bp	0	1	–
g.41736544C > T	CT	−285 bp	0	1	–
g.41736407C > T	CT	−148 bp	1	0	–
g.41736218T > G	TG	+42 bp	1	0	–

*†, variants are located upstream (–) to the transcription start site of TFEB gene at 41736259 (NC_000006.12).*

**TABLE 3:** Clinical and biochemical characteristics of the AMI patients carrying TFEB gene regulatory variants.

Patient no.	Regulatory variant	Sex	Age (years)	TG (mmol/L)	TC (mmol/L)	HDL (mmol/L)	LDL (mmol/L)
1	g.41737274T > C (rs533895008)	M	56	0.84	5.20	1.41	3.20
2	g.41737144A > G	M	66	1.38	3.60	0.80	2.40
3	g.41736987C > T	M	62	1.26	2.83	1.38	2.30
4	g.41736806C > T	M	81	0.94	3.02	1.28	1.60
5	g.41736635T > C (rs975050638)	M	63	1.79	3.99	0.85	2.60
6	g.41736544C > T	M	52	1.36	5.25	0.93	3.60

### Altered Activity of the TFEB Gene Promoter by Regulatory Variants

The effect of the regulatory variants on the transcriptional activity of the TFEB gene promoter was analyzed with a luciferase reporter gene assay. We focused on the regulatory variants identified in AMI patients. The seven regulatory variants only identified in controls or both AMI patients and controls were used as internal controls. Expression constructs containing wild-type and variant TFEB gene promoters pGL3-WT (wild type), pGL3-41377451G, pGL3-41737274C, pGL3-41737144G, pGL3-41737034A, pGL3-41737034C, pGL3-41737005A, pGL3-41736987T, pGL3-41736981G, pGL3-41736806T, pGL3-41736635C, pGL3-41736544T, pGL3-41736407T, and pGL3-41736218G, were transfected into HEK-293 and H9c2 cells. The dual-luciferase activities were measured, and relative activity of wild-type and variant TFEB gene promoters was examined.

**FIGURE 2:**
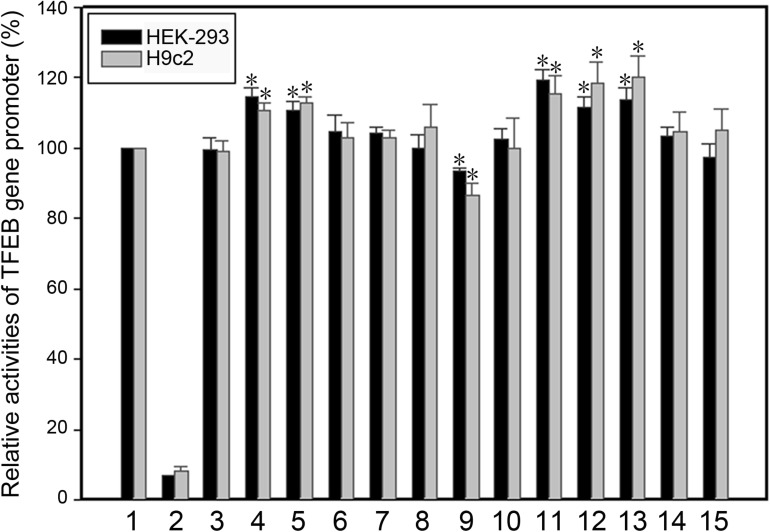
Relative transcriptional activity of wild-type and variant TFEB gene promoters in HEK-293 cells and H9c2 cells. Black bars indicate HEK-293 cells and gray bars H9c2 cells. Empty vector pGL3-basic was used as a negative control. Transcriptional activity of the wild-type TFEB gene promoter was designed as 100%. The relative activity of variant TFEB gene promoters was calculated. Lanes 1, pGL3-WT; 2, pGL3-basic; 3, pGL3-41377451G; 4, pGL3-41737274C; 5, pGL3-41737144G; 6, pGL3-41737034A; 7, pGL3-41737034C; 8, pGL3-41737005A; 9, pGL3-41736987T; 10, pGL3-41736981G; 11, pGL3-41736806T; 12, pGL3-41736635C; 13, pGL3-41736544T; 14, pGL3-41736407T; 15, pGL3-41736218G. WT, wild type. *, *P* < 0.01.

In HEK-293 cells, two regulatory variants (g.41737144A > G and g.41736544C > T) and three SNPs [g.41737274T > C (rs533895008), g.41736806C > T (rs748537297), and g.41736635T > C (rs975050638)] significantly increased the transcriptional activity of the TFEB gene promoter (*P* < 0.01). The SNP [g.41736987C > T (rs760293138)] significantly decreased the transcriptional activity of the TFEB gene promoter (*P* < 0.01). These results indicated that the regulatory variants identified in AMI patients altered the transcriptional activity of the TFEB gene promoter. In contrast, the regulatory variants only identified in controls [g.41737451T > G, g.41737005G > A (rs149166358), g.41736981A > G, g.41736407C > T, and g.41736218T > G] or both AMI patients and controls [g.41737034G > C (rs73733015) and g.41737034G > A (rs73733015)] did not alter the transcriptional activity of the TFEB gene promoter (*P* > 0.05) (Figure 2).

As human cardiomyocyte cell lines are currently not available, the H9c2 rat cardiomyocyte cell line was used. Similar results to that in HEK-293 cells were obtained in H9c2 cells. Two variants (g.41737144A > G and g.41736544C > T) and three SNPs [g.41737274T > C (rs533895008), g.41736806C > T (rs748537297), and g.41736635T > C (rs975050638)] significantly increased the transcriptional activity of the TFEB gene promoter (*P* < 0.01), and the SNP [g.41736987C > T (rs760293138)] significantly decreased the transcriptional activity of the TFEB gene promoter (*P* < 0.01) (Figure 2).

### Regulatory Variant-Affected Binding Sites of Transcription Factors

The binding sites for transcription factors were predicted to be changed by regulatory variants identified in AMI patients. SNP [g.41737274T > C (rs533895008)] may abolish putative binding sites for YB1 (Y-box binding protein 1), MEF2C (myocyte enhancer factor 2C), and MEF2D, and create putative binding sites for MYB (MYB proto-oncogene and transcription factor) and TFCP2 (transcription factor CP2 and also known as LBP1). Variant (g.41737144A > G) may modify the binding site for MYB and the DNA-binding complex of BRCA1 and USF2. The SNP [g.41736987C > T (rs760293138)] may modify the binding sites for SOX18, ZNF75D (zinc finger factor 75D), and ZNF143 factors. SNP [g.41736806C > T (rs748537297)] may abolish the binding sites for SMAD5 (SMAD family member 5), HSF4 (heat shock transcription factor 4), and KLF8 (kruppel-like factor 8), create a germ cell-specific transcription factor ALF-binding site, and modify a KLF6 (kruppel-like factor 6, also known as CPBP) site. SNP [g.41736635T > C (rs975050638)] may abolish a C-MAF (MAF bZIP transcription factor) site, create a RHOXF1 (Rhox homeobox family member 1) site, and modify the binding sites for GATA factors. Variant (g.41736544C > T) may abolish the binding sites for SMAD2 (SMAD family member 2) and P300 and create the binding sites for GATA factors.

### Transcription Factor Binding as Determined by EMSA

**FIGURE 3:**
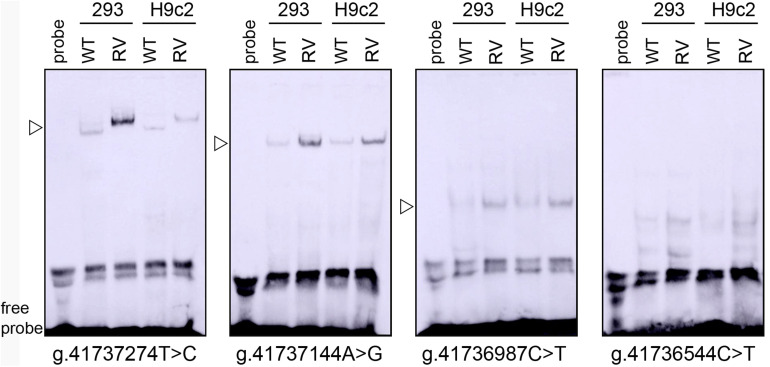
EMSA of biotin-labeled oligonucleotide-containing regulatory variants with nuclear extracts of HEK-293 (293) and H9c2 cells. Free probe was marked at the bottom. The affected binding for an unknown transcription factor was marked with an open arrow. WT, wild type. RV, regulatory variants.

To experimentally investigate whether regulatory variants affected the binding of transcription factors, EMSA was performed with wild-type or variant oligonucleotides (30 bp). The regulatory variants identified in AMI patients were examined. Biotinylated oligonucleotides for the EMSA are shown in Table 4. As shown in Figure 3, the SNP [g.41737274T > C (rs533895008)] abolish the binding of a transcription factor and created a binding site for a new transcription factor. One variant (g.41737144A > G and one SNP [g.41736987C > T (rs760293138)] markedly enhanced the binding of an unknown transcription factor in HEK-293 and H9c2 cells. The affected transcription factor, which acted as a transcriptional activator, requires further identification. The variant (g.41736544C > T) did not affect the binding of transcription factors. Similarly, the effects of other two SNPs [g.41736806C > T (rs748537297) and g.41736635T > C (rs975050638)] on the binding of transcription factors were not detected (data not shown).

**TABLE 4:** The double-stranded biotinylated oligonucleotides for the EMSA.

Variants	Oligonucleotide sequences	Locations
g.41737274T > C	5′-CTGATCTGCTCCAAA (**T/C**)TGGGAGCAGGAGGG-3′	41737289
g.41737144A > G	5′-CTGCTCTGGTGGCTC (**A/G**)GTTGGCCTATGAGC-3′	41737159
g.41736987C > T	5′-CAACGGCTACTGCAC (**C/T**)GTGGGAGTCGAGCC-3′	41737002
g.41736806C > T	5′-TTCCTCTTGACCCTG (**C/T**)ACCCTCCTAGGGCA-3′	41736821
g.41736635T > C	5′-GTGGGTCACTGTGAT (**T/C**)CTAAGAGAAATGGG-3′	41736650
g.41736544C > T	5′-CCTGGAAAGGGAAGA (**C/T**)AGTGGTAGCGCCAT-3′	41736559

## Discussion

To date, many genome-wide association studies have identified a great number of genetic loci for CAD and AMI. However, the collective genetic loci could explain only <10% of cases (Assimes and Roberts, 2016; McPherson and Tybjaerg-Hansen, 2016). Recent studies have suggested that low-frequency and rare genetic variants may confer susceptibility to cardiovascular diseases (Wain, 2014; Sazonovs and Barrett, 2018). Altered TFEB gene expression and subsequent dysfunctional autophagic-lysosomal system have been implicated in human diseases, including cardiovascular diseases (Kuiper et al., 2003; Shen and Mizushima, 2014; Perera et al., 2015; Martini-Stoica et al., 2016). However, TFEB gene expression and regulation have not been characterized in detail. Few mutations or genetic variants in TFEB gene have been reported. In this study, we identified fifteen regulatory variants in the TFEB gene promoter. Among these, six regulatory variants including four SNPs of TFEB gene were identified in AMI patients, which significantly altered the transcriptional activity of the TFEB gene promoter. Three of the regulatory variants evidently affected the binding of unknown transcription factors. In our study population (*n* = 689), TFEB gene regulatory variants were identified in AMI patients with a collective frequency of 0.9% (6/689). Therefore, these TFEB gene regulatory variants may contribute to AMI development as a rare risk factor. In our future work, the effect of downstream autophagic and lysosomal genes of TFEB on AMI will be further explored.

The human TFEB gene has been localized to chromosome 6p21.1. TFEB recognizes the CLEAR motif within its target genes (Carr and Sharp, 1990; Bajaj et al., 2019). TFEB-null mice die at the embryonic stage due to defective placental vascularization (Steingrímsson et al., 1998). Conditional disruption or transgenic mouse models reveal that TFEB has specialized functions in different tissues (Pastore et al., 2016; Perera and Zoncu, 2016; Mansueto et al., 2017; Sergin et al., 2017; Fan et al., 2018). There are five transcript variants of TFEB, and variant two encodes the longest-isoform, promoter region of which was analyzed in this study. To date, the promoter of the human TFEB gene has not been characterized in details. A proximal TFEB gene promoter of 1,600 bp has been reported for its transcriptional activity (Erlich et al., 2018). In human endothelial cells, paternally expressed gene 3 (PEG3) is an upstream transcriptional regulator of the TFEB gene (Neill et al., 2017). There are numerous CLEAR sequences in the TFEB gene promoter, indicating that TFEB regulates its own expression in an autoregulatory loop (Settembre et al., 2013; Nabar and Kehrl, 2017). In response to starvation, TFEB upregulation activates its own transcription, indicating a positive feedback loop that regulates cellular lipid metabolism (Settembre et al., 2013). In this study, we analyzed the proximal promoter of the TFEB gene. The regulatory variants were identified, all of which did not interrupt any CLEAR motif in the TFEB gene promoter. Combined with transcriptional activity assay and EMSA results, SNP [g.41737274T > C (rs533895008)] may create a binding site for a transcription activator. The variants (g.41737144A > G and g.41736544C > T) may enhance the binding of a transcription activator. SNP [g.41736987C > T (rs760293138)] may enhance the binding of a transcription repressor. These transcription factors need to be further identified and investigated.

Under normal conditions, TFEB is located in the cytoplasm. The subcellular localization and activity of TFEB are regulated by its phosphorylation state. TFEB phosphorylation is mediated by several kinases, including mammalian target of rapamycin complex 1 (mTORC1), extracellular signal–regulated kinase 2 (ERK2), glycogen synthase kinase-3β (GSK-3β), and AKT (protein kinase B) (Martina et al., 2012; Settembre et al., 2012; Li et al., 2016; Napolitano and Ballabio, 2016; Palmieri et al., 2017; Vega-Rubin-de-Celis et al., 2017; Puertollano et al., 2018). Protein phosphatase 2A stimulates activation of TFEB by dephosphorylation in response to oxidative stress (Martina and Puertollano, 2018). Phosphorylated TFEB is retained in the cytoplasm, whereas dephosphorylated TFEB translocates to the nucleus to induce the transcription of its target genes. A great number of TFEB direct genes have been identified, which represent essential components of the CLEAR gene network (Palmieri et al., 2011; Perera and Zoncu, 2016). TFEB promotes the gene expression of the autophagy and lysosomes and regulates the lysosomal biogenesis, autophagy, lysosomal proteostasis, lysosomal exocytosis, and lysosomal positioning (Medina et al., 2011; Song et al., 2013; Napolitano and Ballabio, 2016; Perera and Zoncu, 2016; Willett et al., 2017). Moreover, TFEB and TFE3 cooperate in regulating the expression of proinflammatory cytokine genes, controlling the adaptive response of whole-body energy metabolism, and modulating the cellular response to endoplasmic reticulum stress (Martina et al., 2016; Pastore et al., 2016, 2017). Therefore, upregulation or downregulation of TFEB gene expression may lead to dysfunctional autophagy.

Accumulating studies have demonstrated that a window of optimal autophagic activity is critical to the maintenance of cardiovascular homeostasis and function. Excessive or insufficient levels of autophagic flux can each contribute to the pathogenesis of cardiovascular diseases, including AMI (Gatica et al., 2015; Lavandero et al., 2015; Bravo-San Pedro et al., 2017). TFEB is differentially activated in human diseases. In Danon disease, TFEB and downstream targets are activated. Conversely, TFEB is inhibited and an autophagy is blocked in glycogen storage disease type II (Nascimbeni et al., 2017). In this study, the six TFEB gene regulatory variants may lead to TFEB gene upregulation or downregulation, both of which could result in subsequent autophagic dysfunction, contributing to AMI development as a rare risk factor.

In conclusion, fifteen regulatory variants in the TFEB gene promoter were identified in this study, and seven were novel. Among these, six functional regulatory variants in the TFEB gene promoter were identified in AMI patients. Functional analysis revealed that these genetic variants significantly altered the transcriptional activity of the TFEB gene by changing the binding site of unknown transcription factors. These TFEB gene regulatory variants may contribute to AMI development as a rare risk factor. Further studies are needed to investigate these unknown transcription factors and related mechanisms.

## Data Availability Statement

The raw data supporting the conclusions of this article will be made available by the authors, without undue reservation.

## Ethics Statement

The studies involving human participants were reviewed and approved by the Human Ethics Committee of the Affiliated Hospital of Jining Medical University. The patients/participants provided their written informed consent to participate in this study.

## Author Contributions

JZ and BY conceived and designed the experiments and wrote the manuscript. JZ, YZ, XH, and SW performed the experiments. JZ and SP analyzed the data. All authors have read and agreed to the published version of the manuscript.

## Conflict of Interest

The authors declare that the research was conducted in the absence of any commercial or financial relationships that could be construed as a potential conflict of interest.
